# Neofunctionalization of the *Sec1* α1,2fucosyltransferase Paralogue in Leporids Contributes to Glycan Polymorphism and Resistance to Rabbit Hemorrhagic Disease Virus

**DOI:** 10.1371/journal.ppat.1004759

**Published:** 2015-04-15

**Authors:** Kristina Nyström, Joana Abrantes, Ana Margarida Lopes, Béatrice Le Moullac-Vaidye, Stéphane Marchandeau, Jézabel Rocher, Nathalie Ruvoën-Clouet, Pedro J. Esteves, Jacques Le Pendu

**Affiliations:** 1 INSERM, U892; CNRS, UMR6299; Nantes University, Nantes, France; 2 Department of Infectious Diseases, Sahlgrenska Academy, University of Gothenburg, Gothenburg, Sweden; 3 CIBIO, InBIO—Research Network in Biodiversity and Evolutionary Biology, Universidade do Porto, Campus de Vairão, Rua Padre Armando Quintas, Vairão, Portugal; 4 Departamento de Biologia, Faculdade de Ciências da Universidade do Porto, Porto, Portugal; 5 Office National de la Chasse et de la Faune Sauvage, Direction des Etudes et de la Recherche, CS 42355, Nantes, France; 6 Oniris, Ecole Nationale vétérinaire, Agroalimentaire et de L’alimentation, Nantes, France; 7 CITS—Centro de Investigação em Tecnologias da Saúde, IPSN, CESPU, Gandra, Portugal; University of Utah, UNITED STATES

## Abstract

RHDV (rabbit hemorrhagic disease virus), a virulent calicivirus, causes high mortalities in European rabbit populations (*Oryctolagus cuniculus*). It uses α1,2fucosylated glycans, histo-blood group antigens (HBGAs), as attachment factors, with their absence or low expression generating resistance to the disease. Synthesis of these glycans requires an α1,2fucosyltransferase. In mammals, there are three closely located α1,2fucosyltransferase genes *rSec1*, *rFut2* and *rFut1* that arose through two rounds of duplications. In most mammalian species, *Sec1* has clearly become a pseudogene. Yet, in leporids, it does not suffer gross alterations, although we previously observed that rabbit *Sec1* variants present either low or no activity. Still, a low activity *rSec1* allele correlated with survival to an RHDV outbreak. We now confirm the association between the α1,2fucosyltransferase loci and survival. In addition, we show that rabbits express homogenous rFut1 and rFut2 levels in the small intestine. Comparison of rFut1 and rFut2 activity showed that type 2 A, B and H antigens recognized by RHDV strains were mainly synthesized by rFut1, and all rFut1 variants detected in wild animals were equally active. Interestingly, rSec1 RNA levels were highly variable between individuals and high expression was associated with low binding of RHDV strains to the mucosa. Co-transfection of rFut1 and rSec1 caused a decrease in rFut1-generated RHDV binding sites, indicating that in rabbits, the catalytically inactive rSec1 protein acts as a dominant-negative of rFut1. Consistent with neofunctionalization of *Sec1* in leporids, gene conversion analysis showed extensive homogenization between *Sec1* and *Fut2* in leporids, at variance with its limited degree in other mammals. Gene conversion additionally involving *Fut1* was also observed at the C-terminus. Thus, in leporids, unlike in most other mammals where it became extinct, Sec1 evolved a new function with a dominant-negative effect on rFut1, contributing to fucosylated glycan diversity, and allowing herd protection from pathogens such as RHDV.

## Author Summary

There are three members of the α1,2fucosyltransferases gene family in mammalian genomes, *Fut1*, *Fut2* and *Sec1*. The encoded fucosyltransferases are key enzymes for the synthesis of glycans that can be used as ligands by pathogens. However, the polymorphism of expression of these fucosylated glycans on epithelial cell types contributes to protection at the species level. In most mammalian species *Sec1* is a pseudogene and in humans, genetic variation of α1,2fucosylated glycans is provided by *FUT2* polymorphisms. Rabbit haemorrhagic disease virus (RHDV) uses α1,2fucosylated glycans as attachment factors. It induces an acute disease with very high mortalities in rabbit populations. We now confirm an association between genetic markers in the rabbit *Sec1-Fut2* genomic region and survival to RHDV. We show that the *Fut1* gene is the main contributor to the synthesis of RHDV binding sites although individual variation is not achieved by *Fut1* polymorphisms but by variation in levels of *Sec1* transcription. The Sec1 protein acting as a dominant-negative of Fut1, high Sec1 expression leads to a decreased number of RHDV binding sites. Thus, unlike in other mammals, in rabbits *Sec1* underwent neofunctionalization. It contributes to generate diversity of fucosylated glycans, a key mechanism for escaping pathogens such as RHDV.

## Introduction

Following gene duplication the fate of paralogues can be quite variable. Both duplicate copies may be maintained due to a beneficial gene dosage effect or because of functional divergence from the ancestral locus, generating novel gene functions. Alternatively, one of the two duplicates might be driven to pseudogenization because of redundancy with the paralogue [1,2]. In this context, previous analyses of the α1,2fucosyltransferase gene family in mammals indicated that the three members of the family called *Fut1*, *Fut2* and *Sec1* arose by two successive rounds of duplication. The first duplication gave rise to *Fut1* and to the ancestor of *Fut2* and *Sec1*. The second one generated *Fut2* and *Sec1*, these two genes being arranged in tandem in the genome [3,4,5]. Yet, their biological functions are not fully understood. In several mammalian species *Sec1* has become a pseudogene, suggesting functional redundancy with either *Fut1* or *Fut2* [6,7,8,9]. In humans and mice, *Fut1* and *Fut2* are generally not expressed in the same cell types, strongly suggesting functional differentiation [9,10,11]. In accordance, recent data indicate that *Fut1* would be involved in cellular functions such as the development of the olfactory bulb and possibly angiogenesis or adhesion of leukocytes to the vascular endothelium [12,13,14,15], whilst in human, *FUT2* would be involved in a relationship with environmental pathogens including bacteria and enteric viruses [16,17,18,19,20,21]. However, in European rabbit (*Oryctolagus cuniculus*), *Sec1* is not as clearly a pseudogene as in other mammalian species. The coding region of these fucosyltransferases genes is entirely comprised within one exon and it has been observed that in rabbits the *Sec1* exon can encode a full protein with all the characteristics of a fucosyltransferase, albeit with a reduced enzymatic activity compared to those of Fut1 and Fut2 [22,23]. It was later observed that 5 out of 7 *Sec1* alleles encoded proteins devoid of detectable enzymatic activity [23]. This indicated that in European rabbit *Sec1* might still be in the process of pseudogenization, confirming its redundancy as in other mammalian species. Nevertheless, we observed extensive gene conversion involving *Sec1* and both *Fut1* and *Fut2* in rabbits, unlike in several other mammalian species [4]. In those species, including primates, gene conversion could be documented between *Sec1* and *Fut2* only and was limited to the region coding the N-terminal part of the catalytic domain. Since gene conversion leads to homogenization [24], these observations did not fit with the idea that *Sec1* was undergoing pseudogenization in rabbits, but suggested that this gene family was undergoing a specific path of evolution in that species. Interestingly, it was later observed that genetic polymorphisms at the *Sec1* locus are associated with resistance to rabbit haemorrhagic disease virus (RHDV) [23].

RHDV is a member of the genus *Lagovirus* of the *Caliciviridae* family responsible for the rabbit haemorrhagic disease (RHD) that was first reported in China in 1984 [25]. RHD is extremely lethal and highly contagious in domestic and wild adult European rabbits (*Oryctolagus cuniculus*) of both subspecies *O*. *c*. *cuniculus* and *O*. *c*. *algirus* [26]. Resistance to RHD in rabbits below two months of age was readily noticed in the first outbreaks. It was later hypothesized that this could be explained by the weak expression in young rabbits of histo-blood group antigen H type 2 to which RHDV was shown to attach on the epithelial cells of the upper respiratory and digestive tracts, the most likely doors of entry of the virus [27]. Histo-blood group antigens (HBGAs) are carbohydrate structures of glycoproteins and glycolipids that show diversity of expression between individuals. Their function is not completely clear but their location on the most outer part of epithelia suggests that they may be involved in establishing the first contact between hosts and pathogens [16]. In support of this concept various bacterial adhesins, as well as viral capsid proteins, show specificity for these polymorphic glycans and associations between HBGA phenotypes and risk of infection have been described [16,28,29,30]. Human and animal caliciviruses of the Norovirus genus also use HBGAs as attachment factors to infect their host [28]. It appeared that distinct strains of virus bind to different HBGAs structures and that due to the human HBGA polymorphisms, none of these strains can infect the entire population [19]. Because of its high virulence and its documented ability to recognize HBGA structures, RHDV provides a unique model to study the impact of a pathogen on HBGA diversity and reciprocally on the effect of this diversity on the pathogen’s transmission and virulence. However, if the genetic basis of HBGAs diversity in humans is well described, it has been very poorly studied in other mammals, including rabbits, particularly in wild animals.

HBGAs result from the sequential addition of monosaccharides to a precursor glycan chain through specific glycosyltransferases. In particular, synthesis of H type 2 is performed by addition of a fucose residue onto the galactose of the precursor by an α1,2fucosyltransferase. Synthesis can then proceed by addition of either an N-acetylgalactosamine or a galactose in α1,3 linkage to the galactose residue of the precursor to generate the A and B blood group antigens of the ABO system [16]. As mentioned above, there are three α1,2fucosyltransferase genes in mammals: *Fut1*, *Fut2* and *Sec1* and in humans and several other species *Sec1* is a pseudogene. In humans *FUT2* is a highly polymorphic gene with several null or weak alleles undergoing balancing selection [31,32,33]. Homozygous individuals for these alleles possess the nonsecretor phenotype characterized by the absence of blood group A, B, and H antigens in secretions and on various types of epithelial cells. So-called nonsecretor individuals are resistant to various strains of human calicivirus belonging to the Norovirus genus that use α1,2fucosylated ligands for attachment [34]. In this context, it was anticipated that polymorphisms of the rabbit α1,2fucosyltransferases could similarly contribute to control the resistance/susceptibility to RHDV. However, a study on the polymorphisms in the coding sequence (CDS) of rabbit *Fut2* and *Sec1* revealed no effect on Fut2 enzyme activity, whereas for Sec1 either weakly active or inactive enzyme variants were found [23]. Most importantly, Guillon and co-workers found an association between a *Sec1* variant and survival to a devastating RHDV outbreak in a wild rabbit population. This variant encoded a weakly functional enzyme and was always associated with functional Fut2 variants. It was thus suggested that this *Sec1* allele was in linkage disequilibrium with an unknown polymorphism affecting *Fut2* expression since the two genes are located in tandem in the genome [23].

To progress in our understanding of the genetics of HBGAs expression in rabbits and the relationship with resistance/susceptibility to RHDV, we analysed the variation in *Fut1*, *Fut2* and *Sec1* RNA expression in rabbit duodenum tissue, the impact of rabbit *Fut1* CDS polymorphisms on enzyme activity and the relationship between the expression of these three fucosyltransferases genes and virus binding to the duodenum. We unexpectedly uncovered a new mechanism of intra-species production of glycan diversity whereby a paralogue gene (*Sec1*) encodes a protein with a dominant-negative effect on its active isoform (Fut1), corresponding to a neofunctionalization of *Sec1* in rabbits. Further analysis of gene conversion between the three members of the α1,2fucosyltransferase family in mammals suggests that this mechanism is unique to leporids and that the fate of the three members of this gene family diverged between leporids and other mammals.

## Results

### Genetic polymorphisms of the *rFut2* 5’ genomic region and lack of association with mRNA expression

**Fig 1:**
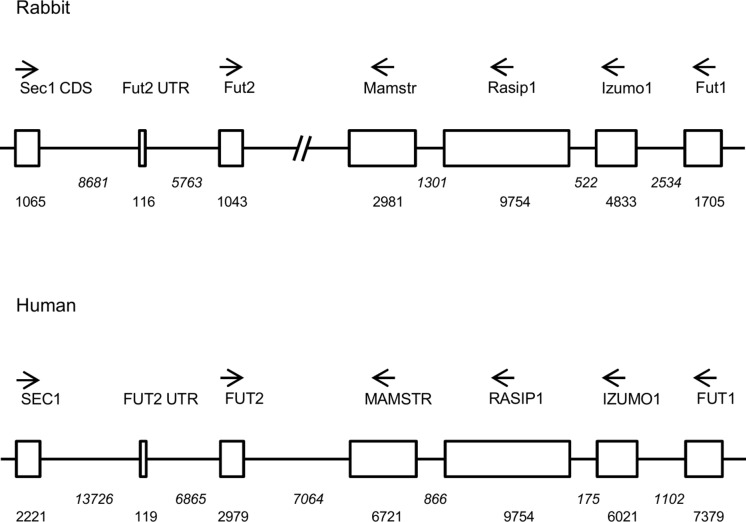
Comparative scheme of the organization of the genome region encompassing α1,2fucosyltransferases genes in European rabbit and human. Genes including coding regions are boxed. Orientation of the genes is given by arrows and distances in nucleotides are given below boxes (in italics for introns and intergenic regions). In the case of *Fut2*, location of the non coding first exon (Fut2 UTR) is given in order to show the distance between its 5’ putative regulatory region and the *Sec1* gene coding determining region (Sec1 CDS).

**Fig 2:**
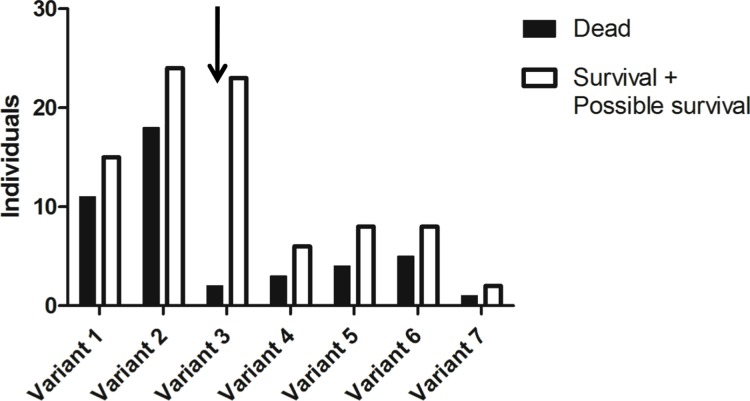
Allele frequencies of the *rFut2* promoter region of dead and survival animals in the 1995–1996 RHDV outbreaks at Chèvreloup, France. The group of survivors may contain a few animals that are adults of the first descendants of survivors rather than direct survivors due to sampling uncertainties (probable survivors). Frequency of the variant 3 allele (arrow) is significantly increased among survivors compared to nonsurvivors (P = 0.002).

It was hypothesized earlier that, in European rabbit, differences in expression of H type 2, a main RHDV ligand or precursor to the A type 2 and B type 2 RHDV ligands, may result from variation in *Fut2* gene transcription rather than enzyme activity [23]. Since the rabbit genome is only partially sequenced, in order to characterize polymorphisms in the promoter region of the rabbit *Fut2* gene (*rFut2*), the genomic region harboring the three α1,2fucosyltransferase genes was first reconstructed from sequences acquired from a genome walking approach and from sequences available in databases. The general organization of this region was found similar to the orthologous human genomic region located on chromosome 19 (Fig 1). Sequencing of 930 bp 5’ of the untranslated first exon of *rFut2* in 65 wild rabbits from Chèvreloup, France, 23 wild rabbits from the Pyrénées Orientales, France, and 29 domestic rabbits, yielded 27 polymorphic positions that segregated into 13 haplotypes, called variants 1–13 (Table 1). Animals from Chèvreloup had been sampled prior to the 1995 and 1996 outbreaks and their survival had been monitored. Variants 1–7 were found in this population. As shown on Fig 2, an association between the presence of variant 3 and survival was observed, confirming the existence of an association between polymorphisms in this genetic region and survival to RHD in wild animals. Indeed, the proportion of rabbits that died during the RHD outbreak differs significantly between variant 3-rabbits (2/25) and other rabbits (42/105) (χ^2^ = 9.23, df = 1, p = 0.002). In order to test if *rFut2* promoter region variant 3 was responsible for a low transcription of the *rFut2* gene, we looked for the relationship between promoter region variants and expression of all three α1,2fucosyltransferase genes by quantitative RT-PCR in the duodenum from a group of 23 wild and 6 domestic rabbits. The RT-PCR data were normalized against two house-keeping genes, GAPDH and β-actin, giving similar results. There was no relationship between *rFut2* expression and the presence of the promoter variant 3 (v3) or any other variant. Indeed, relative expression of *rFut2* mRNA normalized to GAPDH in v3 positive rabbits and v3 negative rabbits were 944 +/- 240 and 612 +/- 90, respectively and normalized to β-actin 143 +/- 33 and 157 +/- 49, respectively. These data indicate that the v3 promoter variant did not affect *rFut2* gene transcription, which invalidated the hypothesis.

**Table 1:** Nucleotide variation found in French wild and laboratory animals in the putative promoter region of rFut2 and inferred haplotypes.

Nucleotide position																											
-811	-752	-741	-691	-684	-680	-676	-670	-660	-542	-501	-466	-462	-456	-433	-377	-376	-365	-231	-211	-162	-150	-146	-26	41	55	57	Haplotype
**A**	**G**	**T**	**G**	**G**	**A**	**A**	**G**	**A**	**T**	**C**	**T**	**T**	**A**	**G**	**C**	**A**	**G**	**G**	**A**	**C**	**C**	**C**	**A**	**C**	**C**	**C**	3 (ref seq) [37]
.	.	.	.	.	.	.	.	.	.	.	C	G	.	.	.	G	.	.	.	T	.	.	.	T	.	.	1 [42]
.	.	.	.	.	.	.	.	.	.	.	.	G	.	.	.	.	.	.	.	.	.	.	.	.	.	.	2 [92]
.	A	.	.	.	.	.	.	.	C	T	C	G	.	.	.	G	.	.	.	T	.	.	.	.	.	.	4 [17]
.	.	C	T	A	C	G	A	G	C	.	C	G	T	A	G	G	.	A	.	.	.	A	.	.	.	T	5 [15]
G	.	C	.	.	.	.	.	.	C	.	C	G	C	.	G	G	A	.	.	.	.	.	.	.	.	.	6 [17]
.	.	C	T	A	C	G	A	G	C	.	C	G	T	A	G	G	.	A	.	.	.	A	.	.	.	.	7 [3]
.	.	.	.	.	.	.	.	.	C	.	.	G	.	.	.	.	.	.	.	T	.	.	.	T	.	.	8 [2]
.	.	.	.	.	.	.	.	.	C	.	.	G	.	.	.	.	.	.	.	.	T	.	.	.	.	.	9 [3]
.	.	.	.	.	.	.	.	.	.	.	.	G	.	.	.	G	.	.	.	.	.	.	.	.	T	T	10 [2]
.	.	.	.	.	.	.	.	.	C	.	C	G	.	.	.	.	.	.	G	.	.	.	G	.	.	.	11 [1]
.	.	C	T	A	C	G	A	G	C	.	C	G	T	.	G	G	.	A	.	.	.	A	.	.	.	T	12 [2]
.	.	.	.	.	.	.	.	.	C	.	.	.	.	.	.	.	.	.	.	T	.	.	.	.	.	.	13 [1]

930 bases upstream of the first untranslated exon of *rFut2* were sequenced from 88 wild and 29 laboratory animals. Positions are numbered from the start codon, where the positive positions are located in the 5’UTR. Dots (.) represent identity with the v3 allele used as a reference sequence. The number of alleles representing each haplotype is given in brackets **[]**. Among laboratory animals, only the v1, v2, v3 and v5 haplotypes were found.

### *rFut1* is the main contributor to RHDV binding sites in the rabbit gut

**Fig 3:**
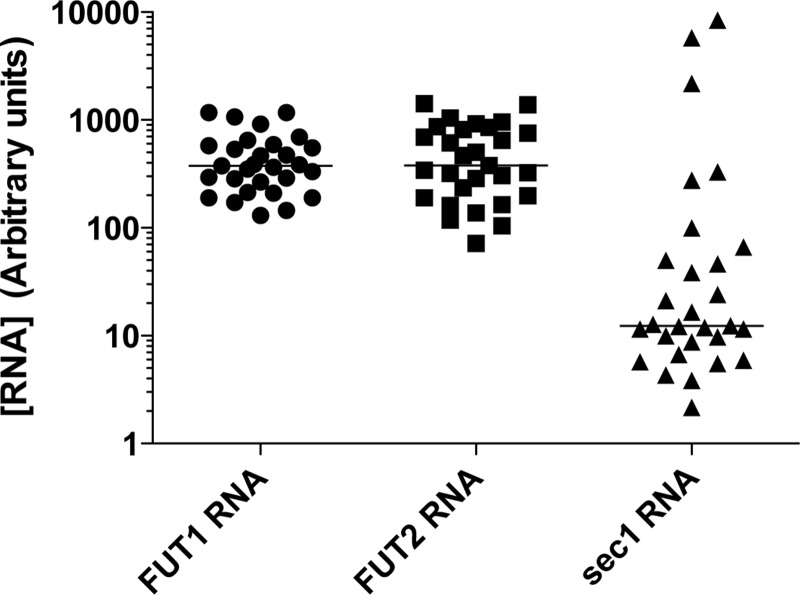
RNA expression of the α1,2fucosyltransferases genes in the rabbit duodenum. Total RNA from duodenum scrapings of individual animals was analyzed using quantitative real-time RT-PCR. Each circle, square, or triangle represents RNA concentration of *Fut2*, *Fut1* and *Sec1* of individual animals normalized to GAPDH. The bars indicate median values for each gene.

**Fig 4:**
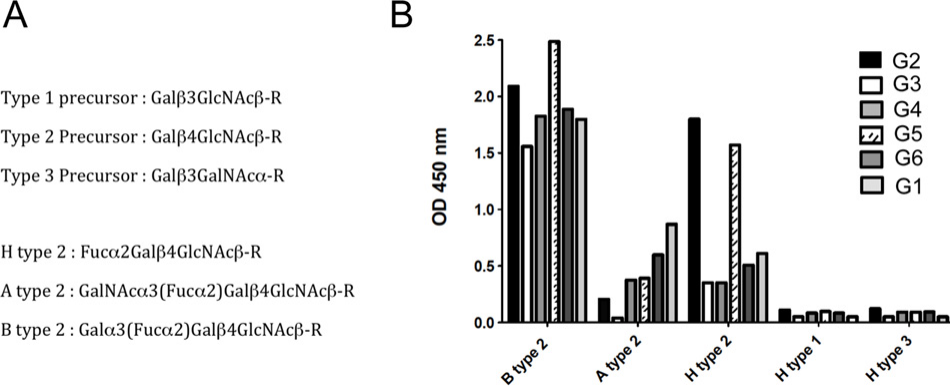
Binding of RHDV strains to immobilized synthetic oligosaccharides. RHDV liver extracts containing 2x10^9^ viral RNA copies, determined by quantitative real-time RT-PCR as previously described, were incubated on immobilized neoglycoconjugates. Binding was detected using primary antibodies against the RHDV capsid protein and HRP-conjugated secondary reagents as described in the materials and methods section. Structures of the types 1, 2 and 3 precursors from which A, B and H structures are built upon addition of monosaccharides. Structures of the H type 2, A type 2 and B type 2 epitopes based on the type 2 precursor are also shown (A). Binding to B type 2, A type 2 and H type 2 of the different RHDV strains is shown as OD 450nm values. No binding was observed to H type 1 or H type 3 (B).

Quantitative RT-PCR analysis of *rFut1* and *rFut2* expression indicated that the two genes were expressed at similar levels with variation between individuals in the 10-fold range (Fig 3). This was unexpected since it had been reported earlier that *rFut1* was not expressed in the gut [35]. In order to determine which gene, of *rFut1* and *rFut2*, contributes the most to RHDV binding sites, we first tested the binding of six distinct RHDV strains, representative of the diversity of RHDV, to a set of immobilized synthetic oligosaccharides of the HBGA family. As shown on Fig 4, the six strains bound to various degrees to either H, A or B type 2 structures. However, no binding was observed to either type 1 or type 3-based A, B or H structures as previously observed [36]. Therefore H type 2, but not H type 1 or H type 3, is either a preferred ligand or the precursor to a preferred RHDV ligand (A type 2 or B type 2). Since it has been previously shown that the human FUT1 enzyme shows some preference for the type 2 precursor over the types 1 or 3 precursors whereas the opposite is true for the human FUT2 enzyme [37,38,39], we sought to determine whether similar preferences for the acceptor substrates would also exist for the rabbit Fut1 and Fut2 enzymes and whether some *rFut1* polymorphisms could contribute to a low expression of RHDV ligands.

**Fig 5:**
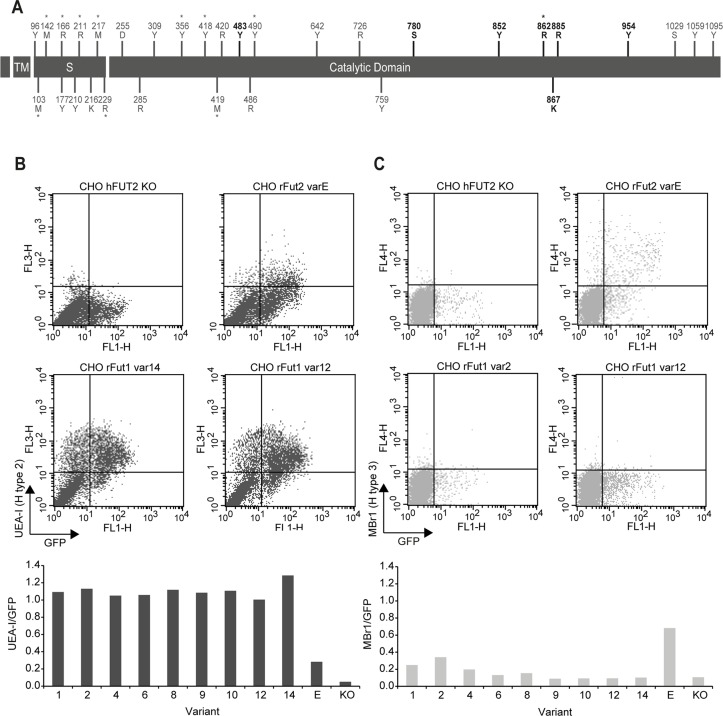
(A) Distribution of *rFut1* polymorphisms along the coding region. The protein domains are represented by black boxes (tm, transmembrane domain; S, stem region). Nucleotide variations are shown above boxes (R = A or G; Y = C or T; K = G or T; S = G or C; M = A or C; D = A, G or T). Amino acid variations are shown below boxes. Positions numbering begins at the start codon and first methionine respectively. The positions in bold refer to the polymorphic positions shared by Sec1, Fut2 and Fut1 (positions 780, 852, 862, 867 and 885) and to the polymorphic positions shared by Fut2 and Fut1 (positions 483 and 954). Asterisks represent non-synonymous substitutions. **(B; C) Flow cytometry analysis of wild rabbit *rFut1* variants**. Enzymes were produced by transfection in CHO cells as N-term GFP fusion proteins. Synthesis of H type 2 and H type 3 was determined using the UEA-I lectin (**B**) and the Mbr1 mAb (**C**), respectively. Transfection of a human null allele (hFUT2 KO) fused to GFP provided a negative control and transfection of the rFut2 allele variant E corresponding to the rFut2 reference sequence provided a positive control. The upper panels show examples of results obtained with two selected rFut1 variants. The lower panels show the ratios between the percentages of UEA-I (H type 2) or Mbr1 (H type 3) positive cells and the percentage of GFP positive cells. In each case, the activity of rFut1 variants is compared to that of the reference rFut2 (variant E).

The *rFut1* coding sequence was thus obtained from 41 animals from France (Chèvreloup n = 19, Cerisay n = 22) and 27 animals from Portugal (Pancas n = 15, Mértola n = 5, Santarém n = 7), yielding a total of 33 polymorphic positions when compared to the rabbit *Fut1* reference sequence (GenBank accession number X80226) (Fig 5A). All 68 individuals presented a silent mutation compared to the reference sequence, which for that reason is not displayed on the figure. Three of the polymorphic positions, with one being non-synonymous, were found in the French populations. In the Portuguese populations, 10 mutations out of 30 were non-synonymous. The mutations were distributed almost uniformly along the coding sequence, although the non-synonymous substitutions were mainly located toward the 5’ end. Five polymorphisms were shared with those previously described for *rFut2* and *rSec1*, while two where shared only with *rFut2* [23]. According to the amino acid variation, two and eleven haplotypes (or variants) were inferred for the French and Portuguese populations, respectively (Table 2). No shared variants were detected between these two groups of populations. In Portugal variants 4, 8, 9, 12 and 14 were rare (frequency<0.02), variants 3 and 10 had a frequency of 0.037, variant 11 a frequency of 0.055, variant 7 a frequency of 0.11, variant 6 a frequency of 0.17 and variant 5 a frequency of 0.5. Variant 2 was the most common in France with a frequency of 0.9. For amino acid position 140, three different amino acids were detected: S, P and Q. The low genetic diversity observed for the French populations when comparing with the Portuguese populations is in accordance with what has been shown for other genetic markers [40,41,42].

**Table 2:** Fut1 amino acid variation found in the Portuguese and French rabbit populations and the inferred haplotypes.

	Amino acid position (nucleotide position)*												
	35	48	56	71	73	77	119	136	140	164	288		
Ref Seq	N	P	V	A	R	P	A	N	S	F	S	Haplotype	Frequency
**France**	.	.	.	.	S	.	.	D	.	.	.	1	0.1000
	.	.	.	.	.	.	.	D	.	.	.	2	0.9000
**Portugal**	.	T	I	.	.	.	.	D	P	L	.	3	0.0370
	H	.	.	.	.	.	.	D	Q	.	.	4	0.0185
	.	T	.	.	.	.	.	D	P	L	.	5	0.5000
	.	.	.	.	.	.	.	D	P	L	.	6	0.1667
	.	.	.	.	.	.	.	D	P	.	.	7	0.1111
	H	.	.	.	.	.	.	D	P	.	.	8	0.0185
	.	.	.	.	.	S	.	D	P	L	.	9	0.0185
	.	.	.	.	.	.	.	D	P	L	G	10	0.0370
	.	T	.	.	.	.	.	D	P	.	.	11	0.0556
	.	.	.	T	.	.	V	D	P	L	.	12	0.0185
	.	T	.	.	.	.	.	D	.	.	.	14	0.0185

*these correspond to the non-synonymous polymorphisms described in Fig 5. Positions are according to the rabbit Fut1 reference (RefSeq; Genbank accession number X80226) and dots (.) represent identity with the reference sequence.

After cloning in an expression vector, rFut1 variants 1, 2, 4, 6, 8, 9, 10, 12 and 14 were tested for their catalytic activity. Variants 5 and 7 were not tested since the amino acid variation described above was almost completely covered by the other variants. Only variation at amino acid position 56 (V/I) was not included as it is located in the stem region and thought to be highly conservative. Variant 11 was not tested since cloning of the full coding sequence failed despite several attempts. Following transfection into CHO cells, the presence of fucose in α1,2 linkage was measured by flow cytometry using the UEA-I lectin that detects H type 2 and a monoclonal antibody (MBr1) that detects H type 3. CHO cells do not express type 1 precursor and therefore synthesis of H type 1 was not measured. The presence of a GFP tail in the recombinant enzymes allowed for control and normalization of transfection and protein expression efficiencies. Synthesis of H type 2 was similar for all variants tested and much higher than for the rFut2 enzyme (rFut2 variant E) used as a positive control (Fig 5B). By contrast, synthesis of H type 3 was either low or undetectable, whereas it was clearly visible for the rFut2 control (Fig 5C). Since, as described above, rFut1 and rFut2 mRNA are expressed at similar levels in the duodenum, these results indicate that rFut1 should be the main contributor to H type 2 synthesis and therefore to RHDV binding sites.

### *rSec1* gene expression is associated with the level of RHDV binding to the duodenum

Quantitative RT-PCR indicated that on average *Sec1* mRNA levels were much lower than those of *rFut1* and *rFut2* in individual rabbits (p<0.0001). However, the 10-fold variation in *rFut1* and *rFut2* mRNA expression appeared quite limited compared to the extreme individual variation found in *rSec1* mRNA levels that varied over 1000-fold (Fig 3).

**Fig 6:**
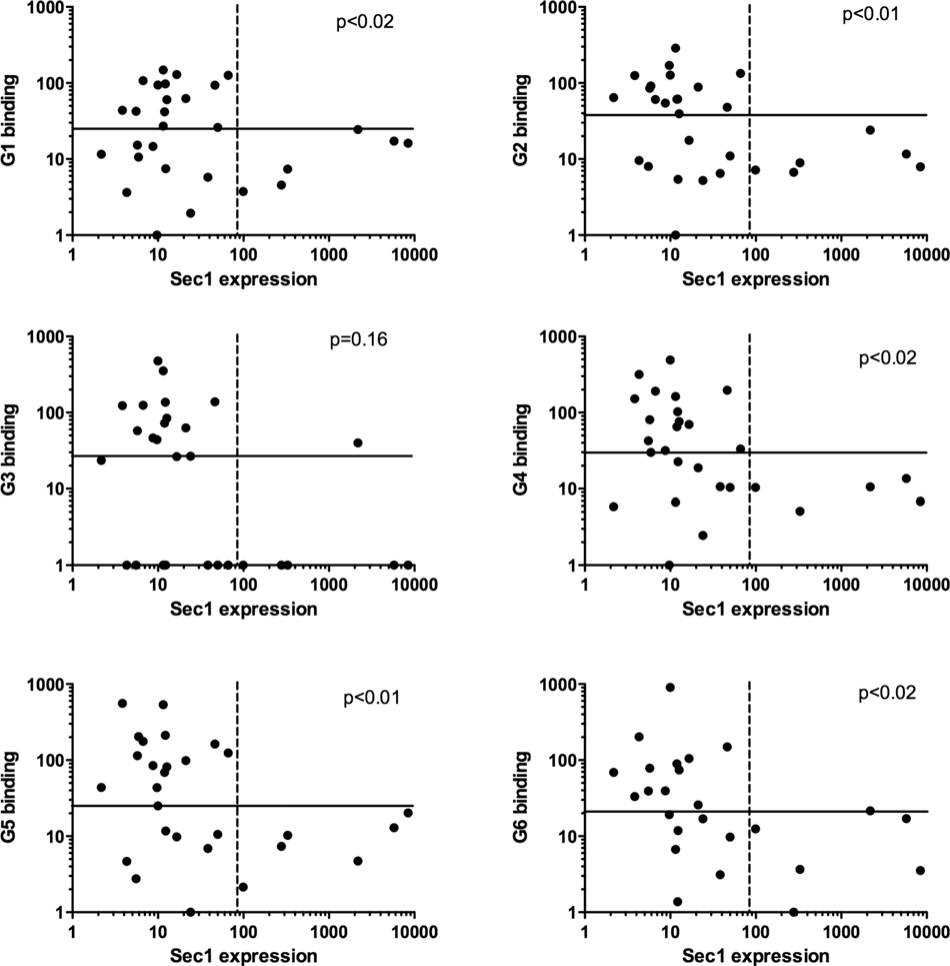
Relationship between expression of *rSec1* RNA and recognition of individual rabbits by RHDV strains. Binding to individual duodenum extracts of six strains of RHDV representative of the virus diversity was determined as described in the materials and methods section. *rSec1* RNA expression in the duodenum of the same rabbits corresponds to the values shown in Fig 3. Plain lines separate high and low RHDV binders defined as above or below median values. Dashed lines separate high and low expressors of rSec1 mRNA defined as animals being above or below the *Fut1* and *Fut2* range of expression. For each RHDV strain P values were obtained from analysis of these categories by Fisher’s exact test. Treating values as continuous variable and testing correlations between rSec1 RNA expression and RHDV binding using the Spearman correlation test also yielded significant relationships (p<0.05) for G2, G3, G4 and G6.

In order to determine whether transcription of the α1,2fucosyltransferase genes could be related to the presence of RHDV binding sites, attachment of the virus to the duodenum extracts was determined in the same group of animals. To this aim, six RHDV strains representative of each virus genotype were used [36]. No relationship was observed between either *rFut1* or *rFut2* expression. However, there was a clear relationship between *rSec1* mRNA expression and RHDV binding (Fig 6). Thus, all animals classified as high *rSec1* mRNA expressers were among the poor binders of most RHDV strains.

### *rSec1* inactive protein acts as a dominant-negative of *rFut1*

**Fig 7:**
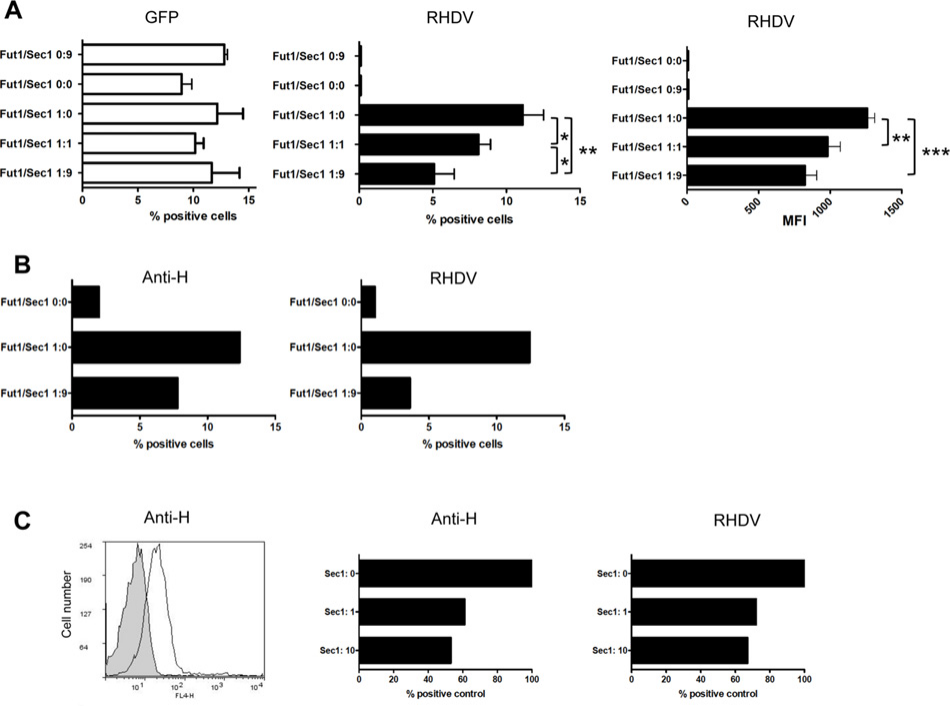
Assay of rSec1 expression on rFut1 activity. CHO cells were co-transfected with a total amount of 10 μg of DNA from an expression plasmid encoding either the Sec1-GFP fusion protein (9 μg) and the truncated hFUT2-GFP (1 μg): Fut1/Sec1 0:9; the truncated rFut2-GFP alone (10 μg): Fut1/Sec1 0:0; the rFut1-GFP fusion protein (1 μg) and the truncated rFut2-GFP (9 μg): Fut1/Sec1 1:0; the rFut1-GFP plasmid (1 μg), the rSec1-GFP plasmid (1 μg) and the truncated rFut2-GFP (8 μg): Fut1/Sec1 1:1; the rFut1-GFP plasmid (1 μg) and the rSec1-GFP plasmid (9 μg). Forty eight hours after transfection, binding of a G2 RHDV strain was determined by flow cytometry. Percentage of positive cells recorded on the FL1 channel for GFP, the FL4 channel for RHDV binding (among total cells) and mean fluorescent intensities of RHDV positive cells are shown. Values represent means and S.D. from 3 independent experiments. * P<0.05; **P<0.01, ***P<0.002 (**A**). Transfection of CHO cells with the truncated rFut2-GFP alone (10 μg): Fut1/Sec1 0:0; the rFut1-GFP fusion protein (1 μg) and the truncated rFut2-GFP (9 μg): Fut1/Sec1 1:0; the rFut1-GFP plasmid (1 μg), the rSec1-GFP plasmid (1 μg) and the truncated rFut2-GFP (8 μg): Fut1/Sec1 1:1; the rFut1-GFP plasmid (1 μg) and the rSec1-GFP plasmid (9 μg): Fut1/Sec1 1:9. Forty-eight hours after transfection, binding of either an anti-H antibody (12.4-LE) or a G2 RHDV strain were determined by flow cytometry. Data represent mean % positive cells from an experiment performed in duplicates (**B**). Flow cytometry plot of the anti-H antibody staining of stably rFut1-transfected CHO cells (negative control in grey). Transfection of rSec1 into stably rFut1-transfected cells. Cells were transfected with either truncated rFut2-GFP (10 μg): Sec1:0; truncated rFut2-GFP (9 μg) and Sec1-GFP (1 μg): Sec1:1; or Sec1-GFP(10 μg): Sec1:10. Forty-eight hours following transfection binding of the anti-H and of RHDV were assessed as above. Results are shown as % of positive cells relative to the negative controls performed in absence of primary antibody or of virus from an experiment performed in duplicates (**C**).

**Fig 8:**
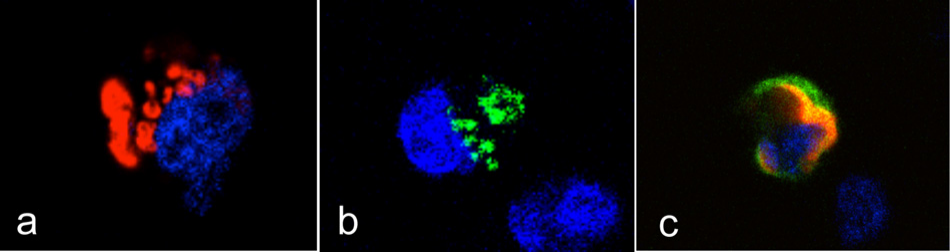
Intracellular localization of the rFut1 and rSec1 proteins. CHO cells were transfected with either rFut1 with a DSred tag (a), rSec1 with a GFP tag (b), or both (c). Images of representative cells expressing either protein alone or co-expressing both proteins are shown. Nuclei are stained in blue.

Since high expression of rSec1 mRNA was related to alleles encoding an inactive enzyme and to low binding of RHDV, we reasoned that the catalytically inactive rSec1 protein might impair synthesis of RHDV binding sites (A, B or H type 2). As described above, rFut1 appears to be the main enzyme controlling for synthesis of A, B and H type 2 in the rabbit duodenum, thus we analyzed the expression of RHDV binding sites at the surface of CHO cells co-transfected with rFut1 and an inactive rSec1 variant. In absence of rFut1 transfection no RHDV binding sites were detected on CHO cells, consistent with their lack of endogenous α1,2fucosyltransferase activity (Fig 7). rFut1 and rSec1 were fused to a GFP at their N-terminus in order to control for the efficacy of transfection and of protein synthesis. In addition, transfections were performed using the same total amount of plasmid DNA encoding for either rFut1-GFP, rSec1-GFP or a truncated inactive rFUT2-GFP fusion protein in order to maintain a constant amount of translated GFP-fusion protein. Under all conditions, GFP was expressed at similar levels and transfection of Sec1 alone did not generate RHDV binding sites. By contrast, transfection of Fut1 alone allowed synthesis of RHDV binding sites since strong RHDV binding was observed in over 10% of the total population of transiently transfected cells. Interestingly, we observed that in the presence of rSec1, synthesis of RHDV binding sites by transfected rFut1 was significantly decreased. The effect was amplified when a higher amount of rSec1 than of rFut1 encoding plasmid was used. Indeed, a 50% decrease in percentage of RHDV binding cells as well as a decrease in their intensity of fluorescence was observed when a ratio of rFut1/rSec1 plasmids of 1:9 was used in the transfection experiment (Fig 7A). To confirm that the decreased RHDV binding was due to a decreased synthesis of H type 2 ligands, the same experiment was performed using an anti-H type 2 antibody. In the presence of rSec1, both the anti-H type 2 and RHDV cell labeling were decreased compared to those of cells transfected with rFut1 alone (Fig 7B). To further confirm these results, we then generated stable transfectants of rFut1. These cells express H type 2 epitopes and upon transfection of rSec1, the percentage of cells expressing the H epitope and attaching RHDV were clearly decreased (Fig 7C). The loss of H type 2 RHDV-binding sites was only partial, likely because only a fraction of the cells was actually transfected by rSec1. These results indicate that the presence of an inactive rSec1 protein blocks synthesis of H type 2 ligands by rFut1. In order to determine how rSec1 blocks the activity of rFut1, we performed confocal microscopy experiments to observe the intracellular localization of these enzymes. This type of glycosyltransferases is known to be expressed in the Golgi apparatus [43]. We observed that cells transfected with rFut1 containing a DSRed tag in N-terminal position showed a typical Golgi-like perinuclear labeling. Cells transfected with a GFP-tagged rSec1 were labeled in a less well-defined cellular compartment that also may be compatible with the Golgi. Strikingly, all cells doubly expressing rFut1 and rSec1 showed a cytoplasmic labeling with both the red (rFut1) and the green (rSec1) tags, clearly indicating that the presence of rSec1 displaced rFut1 out of the Golgi to the cytoplasm, explaining the impairment of its function (Fig 8).

### Evolution of the α1,2fucosyltransferases genes in leporids: A particular gene conversion pattern

A limited and most likely ancient gene conversion between *Sec1* and *Fut2* in primates and several other mammalian species has been described earlier [4]. In these species gene conversion was restricted to the gene fragment coding the N-terminal domain of the catalytic region. Since in the same species *Sec1* is a pseudogene presenting clear coding-sequence alterations we concluded that gene conversion with either *Fut1* or *Fut2* would likely no longer be observed because of deleterious effects on the Fut1 and Fut2 enzymes. However, we additionally observed that in European rabbit, the three α1,2fucosyltransferases genes are involved in gene conversion all along the catalytic domain coding sequences. This observation led us to hypothesize that in rabbits, *Sec1* might have acquired a function distinct from those of the two other α1,2fucosyltransferases genes. The results presented above indicating that rSec1 acts as a dominant-negative of rFut1 are fully consistent with its neofunctionalization in rabbits. In order to determine if gene conversion involving the three members of the α1,2fucosyltransferase gene family could also be observed in other species, which would be consistent with the acquisition of a new function in Sec1, we analyzed the sequences of the three genes (*Fut1*, *Fut2*, *Sec1*) in 23 mammalian species including six genera of leporids.

**Fig 9:**
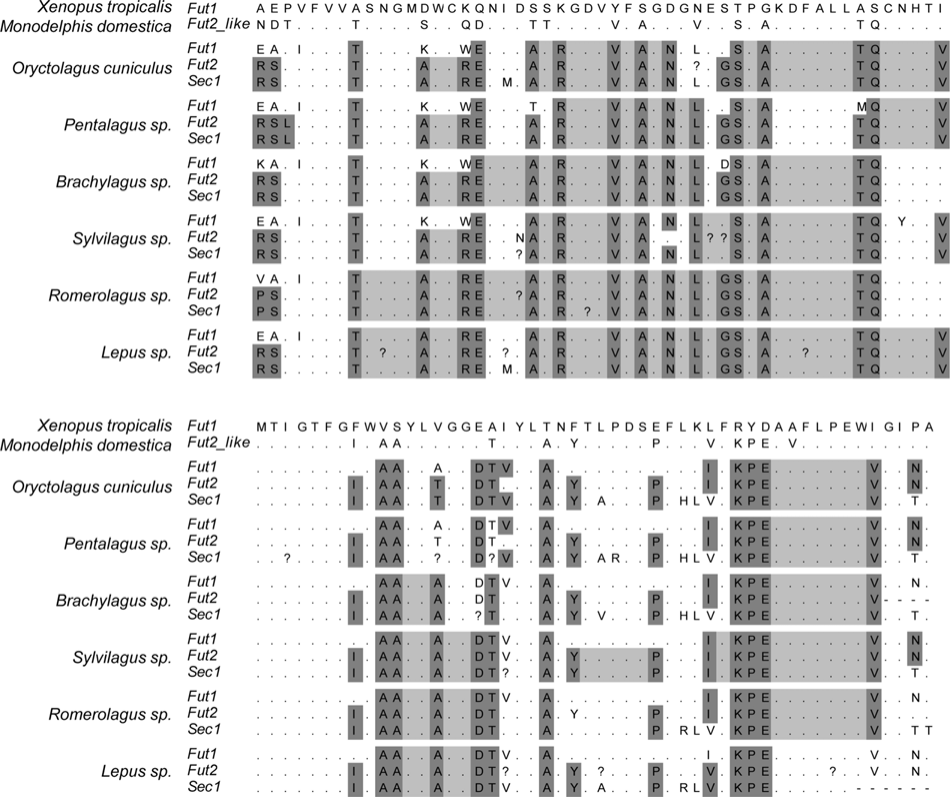
Alignment of the catalytic domain of α1,2fucosyltransferases for 6 leporid species illustrating the gene conversion events. For each species the 3 enzymes are shown. Dark grey: positions that differ in more than one gene relatively to *Xenopus tropicalis*. Light grey: positions that are identical in more than one gene between the positions identified by dark grey. Black: premature stop codons. Dots mean identity with the *X*. *tropicalis* sequence.? indicate positions that were ambiguous upon sequencing. The upper part of the alignment comprises amino acid positions 254 to 304 of *Homo sapiens* FUT1, the lower part comprises amino acids positions 305 to 354 of *Homo sapiens* FUT1. Full names of each species and accession numbers of sequences are given in Supplementary Table 1.

Visual inspection of the alignment of the catalytic domain of the three enzymes in the different mammalian species highlighted major differences in the gene conversion extent, particularly between leporids and the remaining mammals (Fig 9 and S1 Fig). In leporids, amino acid motifs shared between the Fut2 and Sec1 encoded enzymes are visible from the beginning of the alignment and extend up to amino acid position 217. In contrast, for the remaining mammals, motifs are only shared between amino acids 133 and 179. Other Fut2-Sec1 shared motifs are observed, but they extend for a few amino-acids only; in pigs, however, these motifs are larger although Sec1 is a pseudogene. Regarding Fut1-Fut2-Sec1 shared motifs, and although they can be identified for most species, in leporids they extend from amino acid position 262 to 305. In addition, in the 3’ region (amino acid positions 341 to 351), a short fragment of gene conversion can also be observed for leporids (Fig 9). Interestingly, in primates, this region is located immediately downstream of the *Sec1* premature stop codon in gorillas, humans and chimpanzee (S1 Fig).

In order to determine recombination breakpoints, a GARD analysis was performed considering three distinct groups: all mammals, all mammals except leporids and only leporids. This partition was according to the different extent of gene conversion as observed from the amino acid alignment. One recombination breakpoint was consistently detected when analyzing mammals at nucleotide 375 of the alignment. When analyzing leporids only, the recombination breakpoint was detected at nucleotide 501 (Table 3).

**Table 3:** List of the breakpoints detected by GARD and their statistical significance.

Dataset	Breakpoint	ΔAICc^1^	LHS p-value^2^	RHS p-value^3^
All mammals	375	569.377	0.0002*	0.0002*
All mammals except leporids	375	555.17	0.0002*	0.0002*
Only leporids	502	304.557	0.0002*	0.0002*

^**1**^ Akaike Information Criteria (AICc) for b = 0, b = 1 and improvement relative to the model with one fewer breakpoint (ΔAICc).

^2^KH test was used in both directions to compare phylogenies constructed from the alignment segment to the left hand side (LHS^**2**^) and right hand side (RHS^**3**^) of each estimated breakpoint. All *p-values* have been adjusted by Bonferroni correction.

The phylogenetic trees (S2 Fig) constructed for the three genes considering the different dataset partitions and the recombination breakpoints identified by GARD (Table 3) showed a wider extent of gene conversion between *Fut2* and *Sec1* in leporids than in the other groups of mammals analyzed. Although gene conversion events involving *Fut1* are apparent from visual inspection of Fig 9 and S1 Fig, which suggested its involvement in the gene conversion in leporids, they are not clear from the phylogenetic trees.

## Discussion

A major role of α1,2fucosyltransferases, particularly documented for FUT2 in humans, is their contribution to the synthesis of glycan motifs that present common polymorphisms [16]. These carbohydrates expressed on epithelial surfaces in contact with the environment, such as the gut, the upper airways and the lower genito-urinary tract, constitute attachment factors for various pathogens. By restricting transmission of the pathogen their polymorphism provides what may be called “herd innate protection” by reference to “herd immunity” or indirect immunity whereby the presence of immunized individuals in a population confers protection to immunologically naïve individuals [28]. The earlier observation that a rabbit *Sec1* allele was associated with survival to a devastating RHDV outbreak led to the hypothesis that this allele was in linkage disequilibrium with a *rFut2* promoter allele responsible for low expression of the corresponding Fut2 enzyme. This was justified by the observation that although rabbits express highly variable levels of A, B or H antigens that require an α1,2fucosyltransferase for synthesis, all allelic variants of rFut2 presented equal enzymatic activities [23]. Thus individual differences in rFut2 transcription rather than in enzyme catalytic properties could have accounted for large differences in the expression of the A, B or H glycan motifs. Sequencing of the rFut2 promoter region revealed an association between a genetic variant and survival to RHDV in a wild rabbit population. However, this promoter variant could not be associated with an altered transcription level of *rFut2* and therefore it is unlikely that a low transcription of *rFut2* could be responsible for resistance to RHDV. Unexpectedly, we observed that in the small intestine mucosa of rabbits, unlike in humans, *rFut1* transcription was as high as that of *rFut2*. The six RHDV strains tested, spanning the genetic diversity of RHDV, recognized carbohydrate motifs based exclusively on the type 2 precursor. We observed that rFut1 enzyme variants were all equally active, generating H type 2 motifs but not H type 3 motifs. By contrast, the rFut2 enzyme preferentially generated H type 3, indicating that rFut1 is the main contributor to the synthesis of RHDV ligands. This is at variance with the synthesis of HBGAs in the human small intestine that is essentially dependent upon FUT2 and HBGAs based on type 1 precursor [11].

The mRNA levels of rFut1 and rFut2 were relatively homogeneous and were not related to RHDV binding. Therefore the wide individual variation in α1,2fucosylated motifs used as attachment factors by RHDV cannot be accounted for by either rFut1 or rFut2 allelic enzyme variants of catalytic activity, or by allelic variants of transcription. How then is the individual variation generated?

Compared to the expression of *rFut1* and *rFut2* transcripts, that of *rSec1* was much lower for most animals, apparently consistent with the lack of enzyme activity and evolution toward a complete loss of function. Nevertheless, in a small fraction of animals, *rSec1* mRNA levels were in the same range or even higher than the *rFut1* and *rFut2* mRNA levels and this elevated *rSec1* expression was associated with low RHDV binding. This suggested that the rSec1 protein could function as a dominant-negative of the rFut1 enzyme and in support of this hypothesis we observed that co-transfection of rSec1 decreased the level of RHDV binding sites synthesized by rFut1. Although it is not clear at present how rSec1 expression represses rFut1 activity, it has been shown that various glycosyltransferases, including fucosyltransferases function as dimers or oligomers [44,45,46]. Furthermore, a report indicated that human FUT1 transfected in CHO cells is exclusively present as dimers [47]. Dominant-negative effects of artificially introduced mutations that inactivate the enzyme activity have been observed for several glycosyltransferases and a dominant-negative function of an inactive splice variant isoform of a glucuronyltransferase was demonstrated [48,49,50,51]. It is therefore reasonable to hypothesize that enzymatically deficient rSec1 could form dimers with rFut1, leading to a decrease of rFut1 catalytic ability. Further studies will be required to understand the molecular mechanism underlying the rSec1 dominant-negative function.

Overall our data indicate that in rabbits there are no functionally significant individual variations in rFut1 or rFut2. Instead, variations in levels of rSec1 transcription contribute to generate individual differences in the degree of α1,2fucosylation of the gut mucosa, leading to differences in recognition by at least one dreadful pathogen. Nevertheless, since not all animals classified as low binders of RHDV expressed high levels of *rSec1* RNA, additional factors must contribute to the variation in RHDV attachment. We previously reported that A and B antigen expression were contributing to modulate binding in a strain-specific manner [36]. Indeed, the ability of individual RHDV strains to recognize A, B and H type 2 motifs is highly variable and the expression of the A or B epitopes are differentially expressed between rabbits. Moreover, some strains such as G1 strains preferentially recognize the difucosylated motif Lewis y. Synthesis of this motif requires an α1,3fucosyltransferase in addition to an α1,2fucosyltransferase. Individual variation in the levels of α1,3fucosyltransferase in the rabbit gut may also exist. Understanding the genetic determinisms of these additional glycan modifications of rabbit gut mucosa will require further investigation. Moreover, other unknown factors may contribute to generate individual variation in the repertoire of rabbit gut glycans recognized by RHDV.

The genomic region where the three α1,2fucosyltransferases genes lie presents the same organization in rabbits and humans with the *Sec1* and *Fut2* genes in tandem and the *Fut1* gene localized further apart and separated from its paralogues by three other genes. Since gene conversion is facilitated by genomic proximity [24] the differences observed between primates and other mammals on the one hand and leporids on the other hand cannot be explained by differences in genomic organization. In leporids extensive conversion between the three members of the gene family appears to take place. This is expected to homogenize these sequences, suggesting that each of the family members retains some functionality. By contrast, in other mammalian species, including primates, gene conversion is limited to a small part of the *Fut2* and *Sec1* coding regions and never involves *Fut1*, consistent with the pseudogenization of *Sec1* and functional divergence between the other two members of the family in these species. Indeed, in species where *Sec1* is a pseudogene, either due to frameshifts, premature stop codons or mutations that impair the enzyme’s activity, the extent of gene conversion is limited to regions where *Sec1* has no deleterious mutations or where it retains its characteristics as α1,2fucosyltransferase. For example, in pig, *Sec1* is a pseudogene due to the lack of the start codon (Meijerink et al 1997), but the remaining putative coding sequence presents no further deleterious mutations allowing to a larger extent of *Fut2-Sec1* gene conversion when comparing to the other species (except leporids).

These observations show that the α1,2fucosyltransferases paralogues have undergone different evolutionary pathways in leporids than in other mammals. The following scenario can be proposed. The first duplication event generated subfunctionalization of the two paralogues *Fut1* and the ancestor of *Fut2* and *Sec1* in all mammals, through expression in different cell types. In humans and likely in most other mammalian species, *FUT1* is involved in cellular functions such as the development of sensory neurons, angiogenesis or leukocytes adhesion [12,13,14,15,52]. Consistent with these roles, the frequency of *FUT1* null alleles is well below 0.01, although not associated with any obvious defects in the homozygous state [53]. As discussed above, with null alleles at a near 0.5 frequency, *FUT2* is involved in herd innate protection due to its common polymorphisms and its expression on epithelial cells in direct contact with microbes [16,28]. For this reason it is strongly under balancing selection [32]. The second round of duplication generated *Fut2* and *Sec1*. Following this duplication, *Sec1* has been redundant in most species and evolved toward pseudogenization and since then it has no longer been involved in gene conversion with its paralogues [4]. This is clear from S1 Fig where clustering of Fut2 and Sec1 together rather than with their orthologues can be observed, although with distinct patterns: in (A), where the phylogenetic relationships were inferred for the entire catalytic domain, for most species, Fut2 and Sec1 cluster apart; in pig, rat and mouse, Fut2 and Sec1 sequences cluster together, as in these species the homogenization due to gene conversion is quite extended when compared to the remaining species; in (B), Fut2 and Sec1 cluster together for more species as the segment used corresponds to the region where gene conversion is most evident; in (C) the pattern resembles the tree in (A) as a consequence of the more extended gene conversion in pig, rat and mouse and the absence of gene conversion in the other mammals. Thus, in mammals other than leporids, gene conversion also occurs, but it is quite limited in extent and most likely has no impact on the enzymes’ activity. Yet, in leporids where gene conversion between *Sec1* and both *Fut1* and *Fut2* has been maintained (Fig 9 and S2D, S2E, and S2F Fig), the Sec1 protein follows a convergent evolution with the other two members of the gene family. It has essentially lost its enzyme activity but has acquired a dominant-negative function of its Fut1 and possibly Fut2 isoforms in tissues where they are co-expressed. In European rabbit, there is no functional polymorphism of either Fut1 or Fut2, but the levels of Sec1 expression contribute to produce glycan diversity required for herd innate protection. Thus, rather than becoming a pseudogene as in other mammals, in leporids, *Sec1* acquired a new function through regulation of the activity of the other α1,2fucosyltransferases.

## Materials and Methods

### Ethics statement

Work involving the acquisition and sampling of French wild rabbits in Chèvreloup and Cerisay was carried out with permission from the bylaw N° 2009–014 issued by the Paris Prefecture to the Office National de la Chasse et de la Faune Sauvage (ONCFS) and the specific permit N° 11/04 to Dr. Stéphane Marchandeau issued by the ONCFS. Rabbits were caught alive in cage traps or with ferrets. The use of laboratory animals was carried out in a group V animal facility (agreement N° 44267), under specific agreement N° 006933 from the National Committee of Ethics on Animal Experiments of the Ministry of Higher Education and Research. Animal care and handling were performed in strict accordance with the recommendations of the French National Guide for the Ethics of Animal Experiments and euthanasia was performed under xylacine and ketamine anesthesia.

### Rabbit samples

For the characterisation of the CDS of rFut1 and rSec1, total genomic DNA was extracted from ear samples collected upon ear tagging of surveyed wild populations in France (Chèvreloup, n = 19 and Cerisay, n = 22) and Portugal (Santarém, n = 7, Mértola, n = 5 and Pancas, n = 15) with the QIAamp DNA Mini kit (Qiagen, Courtaboeuf, France) according to the manufacturer’s protocol. The French population from Chèvreloup experienced two consecutive outbreaks in 1995 and 1996 and samples had been collected prior to the outbreaks in 1994 and 1996 [54,55]. For the characterization of rFut2 5’UTR, 65 DNA samples from the Chèvreloup population were used in addition to DNA obtained from duodenum samples.

Rabbit duodenums were collected from domestic New Zealand White rabbits (29 animals) and wild rabbits from the Pyrénnées Orientales, France that had been freshly killed by hunters (23 animals). The first 5 cm posterior to the gastroduodenal junction was removed after clearing the section from intestinal contents, the sample was vigorously rinsed in phosphate buffered saline (PBS) and stored in RNAlater (Ambion, Life Technologies, Paisley, UK) at -20°C. Sections of the duodenum were then rinsed in PBS, opened and scraped into RLT lysis buffer (Qiagen, Hilden, Germany) containing β-mercaptoethanol. The tissue scrapings were homogenized and split into three parts for ELISA assays, RNA and DNA extraction. The ELISA scrapings were boiled for 10 minutes.

### Reconstruction of the rabbit α1,2fucosyltransferases locus

To locate the 5’UTR of *Fut2*, a RACE was performed on rabbit RNA extracted from duodenum tissue. Ten μg of RNA were used and the RACE was performed according to the manufacturer’s instructions (FirstChoice RLM-RACE kit, Life Technologies, Paisley UK), giving the 118 bp 5’ UTR. Primers were designed in this UTR for a genome walk in the 5’ and 3’ direction. This was performed using the GenomeWalker Universal Kit (Clontech, Mountain View CA) according to the manufacturer’s instructions. These acquired sequences allowed to perform BLAST searches of the trace archives where long matches of at least 400bp were added on in the 5’ and 3’ direction from the UTR as well as in the 5’ and 3’ direction of *Fut2* and *Sec1*, respectively. The 930 bp 5’ of the 5’UTR of *Fut2* were then sequenced for the promoter region of *Fut2*.

### RT-PCR quantification of *Fut1*, *Fut2* and *Sec1* RNA expression

The RNeasy Mini kit (Qiagen) was used for purification of total RNA according to the manufacturer’s instruction. Reverse transcription was performed with 400 ng of RNA, using the Superscript II Reverse Transcriptase (Life Technologies, Paisley, UK) according to the manufacturer’s instructions. Forty ng of cDNA were then used for real-time PCR (Universal Mastermix, Applied Biosystems, Life Technologies, Paisley, UK), also according to manufacturer’s instructions, on a Mx3005P (Stratagene Agilent Technologies, Massy, France). All primers and probes were designed using Primer Express (Applied Biosystems, Life Technologies, Paisley, UK) and plasmids containing the amplicon were used as a standard for each run. Relative concentrations of transcripts from the different α1,2fucosyltransferase genes were determined using the ΔCT method according to manufacturer’s instruction regarding TaqMan chemistry. The rabbit house-keeping genes GAPDH and β-actin were both separately used as normalization standards, linearising the real-time PCR data against house-keeping gene, giving very similar results.

### RHDV binding assay

RHDV binding to rabbit duodenum scrapings or synthetic sugars was analyzed as previously described [36]. Briefly, rabbit duodenum scrapings were coated diluted in a range of dilutions in 0.1M sodium carbonate buffer or 1μg of synthetic sugars was coated in the same buffer. Plates were blocked with 5% non-fat dry milk diluted in PBS or distilled water. RHDV from six strains belonging to genetic groups G1 to G6 as defined in [56] was then added to the plates in similar amounts. To this aim, RHDV strains were prepared from infected liver PBS extracts and quantified by real time RT-PCR using a plasmid containing an RHDV inserted sequence as an internal standard. Binding of RHDV strains was detected using a high-titered rabbit serum Lp4 or an RHDV monoclonal antibody 2G3 for G1–G5 and G6 detection, respectively. Secondary antibodies anti-rabbit conjugated with HRP were used against Lp4 and anti-mouse biotin followed by avidin-HRP was used for RHDV detection with 2G3 anti-RHDVa (G6) monoclonal antibody. TMB (BD Bioscience, San Jose CA) was used as a substrate for all assays and O.D. values were measured at 450nm. To compare binding data between individuals, a threshold was set at 3 times the background. Dilution values of each sample for crossing the threshold were analyzed. All values were normalized against values obtained with the mannose-binding lectin *Concavalin A* to control for differences in the amount of material scraped, as protein quantification proved to be difficult due to the addition of β-mercaptoethanol to remove any potential anti-RHDV antibodies in the duodenum that would interfere with the analysis.

### Amplification and cloning of the open reading frame of the *rFut1* and *rSec1* genes

Amplification of the coding sequences was performed using the AmpliTaq gold kit with GenAmp 10x PCR Gold Buffer and MgCl_2_ Solution (Applied Biosystems, Foster city, USA) with the primers rFut1 ATG 5’ ATGTGGCCTCCGAGCC 3’ and rFut1 STOP 5’ CTATCCCAGAAATCTCCAGGGC 3’ for *rFut1* or rSec1.3 5’ ATGAGATTCGCCCCTGACTA TGTCC 3’ and rSec1.2 inv 5’ CTAGAGGCCACTCCACAAGGC 3’ for *Sec1*. The amplified PCR products were 1122 bp and 1044 bp long, respectively. Cloning was performed either in the pcDNA 3.1 vector using the NT-GFP Fusion TOPO TA Expression kit (Invitrogen, Paisley, UK), or in the pDSred vector (Clontech, Rockville, MD), according to the manufacturers’ instructions. The bacteria used for transformation were *E*. *coli* One Shot TOP10F’ (Invitrogen, Paisley, UK). After controlling for correct orientation of the inserted sequences several clones were sequenced and the resulting correctly oriented sequences generated GFP or DSred fusion proteins with the GFP or DSred tags located at the N-terminus.

### Assay of the α1,2fucosyltransferase activity of *rFut1*

CHO cells were cultured in RPMI 1640 medium supplemented with 10% fetal calf serum, 2 mM L-glutamine, free nucleotides (10 μg/mL), 100 U/mL penicillin and 100 mg/mL streptomycin (Gibco, Paisley, UK). Cells were cultured to confluence after dispersal with 0.025% trypsin and 0.02% EDTA. Following amplification and purification with the Qiagen Miniprep kit, the recombinant pcDNA3.1 constructs were transfected into the CHO cells using ICAFECTIN®448 (In-Cell-Art, Nantes, France) following the manufacturer’s instructions. A stable rFut1 expressing cell line was obtained by transfection of the GFP-rFut1 plasmid followed by selection of the cells using G418 (Promega France, Charbonnières, France). Stable expression of the H epitopes on the selected cells was controlled by flow cytometry using either the UEA-I lectin or the 12-4LE anti-H type mAb. For transient transfections, cells were collected 24 hours after transfection and the presence of α1,2-linked fucose residues was tested by flow cytometry using the UEA-I lectin or the 12-4LE mAb that detect the H type 2 (Fucα 2Galβ4GlcNAcβ-R) motif and the MBr1 mAb that detects the H type 3 (Fucα 2Galβ3GalNAcα -R) motif. To this aim, 2.5 x 10^5^ viable transfected cells were incubated in the presence of either biotin-labeled lectin at 5 μg/mL (Vector labs, Peterborough, UK) or the MBr1 mAb (Alexis Biochemicals, San Diego, CA) at 5 μg/mL for 20 min at 4°C. After three washings with PBS, cells were incubated under the same conditions in the presence of either PerCP-conjugated streptavidin (BD Biosciences, San José, CA) at 0.5 μg/mL or a Cy5-conjugated anti-mouse IgG (BD Biosciences) at a 1/500 dilution. After three more washings with PBS, cell fluorescence was measured on a FACScalibur flow cytometer (Becton-Dickinson) and analysed using the CellQuest program (Becton-Dickinson). The transfected protein expression was quantified by the GFP fluorescence recorded on the FL1 channel. The presence of the H type 2 and H type 3 motifs was detected on the FL3 and FL4 channels, respectively.

Co-transfection experiments were conducted to determine the effect of rSec1 on rFut1 enzyme activity. They were performed in 6-well plates using 10 μg plasmid DNA and 20 μL Lipofectamin 2000 (Life Technologies) according to the manufacturer’s instructions. Positive control of rFut1 activity was obtained by transfecting CHO cells with 1 μg of the pcDNA3.1 plasmid containing an rFut1-GFP construct and 9 μg of the pcDNA3.1-truncated hFUT2-GFP vector. This last construct was designed to allow expression of an inactive enzyme fused to GFP (GFP plasmid). To obtain a 1:1 rFut1/rSec1 ratio, co-transfection was performed with 1 μg rFut1-GFP plasmid, 1 μg rSec1-GFP plasmid and 8 μg truncated rFut1-FP plasmid; to obtain a 1:9 rFut1/rSec1 ratio, 1 μg rFut1-GFP plasmid and 9 μg rSec1-GFP plasmid were used. Forty-eight hours following transfection, expression of RHDV binding sites was determined by flow cytometry. To this aim the transfected cells were incubated for 30 min at 4°C with a 10 μg/mL suspension of virus-like particles from a G2 RHDV strain prepared as described previously [27]. After washings, their binding was detected using the monoclonal 13B5 anti-RHDV diluted at 1.2 μg/mL followed by a Cy5-conjugated anti-mouse IgG (BD Biosciences) at a 1/500 dilution as described above.

### Confocal microscopy analysis

CHO cells were transiently transfected with either 1 μg rFut1-DSred plasmid and 1 μg empty vector, or 1 μg rSec1-GFP plasmid and 1 μg empty vector, or with both rFut1-DSred plasmid and rSec1-GFP plasmid, 1 μg each. Forty-eight hours later, cells were detached and plated on Lab-Tek chamber slides (NUNC) overnight. Following washes with PBS, cells were fixed with 4% paraformaldehyde for 15 min at room temperature, washed with PBS and nuclei were stained using Hoescht 33422 from Molecular Probes (Life Technologies). After PBS washes, slides were mounted with Prolong Gold antifading from Molecular Probes. Fluorescence was observed using a Nikon A1 confocal microscope and image processing performed with ImageJ.

### Gene conversion analyses

In order to explore and fully determine the extent of gene conversion in mammals, the available sequences of the three α1,2fucosyltransferase genes *Fut1*, *Fut2* and *Sec1* from mammals were retrieved from public databases (see S1 Table for accession numbers and gene scaffolds). Only mammals for which sequences of the three genes were available were used in this study. Given the ancestry relatively to other mammals, and thus the closer relation to the ancestral forms of the α1,2fucosyltransferases, sequences from *Monodelphis domestica* and *Xenopus tropicalis* were also included. The family *Leporidae* started to radiate around 14 million years ago [57]. This family is composed by 11 genera. In this study, gDNA samples from five genera were amplified and sequenced for the three genes: *Romerolagus diazi*, *Lepus spp*., *Brachylagus idahoensis*, *Sylvilagus spp*. and *Pentalagus furnessi*. These genera diverged from *Oryctolagus* at 14, 12, 10, 10 and 9 million years, respectively [58,59]. For amplification of *Fut1*, a combination of two pairs of primers was used. For the first pair, Fut1ATG (5' ATGTGGCCTCCGAGCC 3') and Fut1conservR (5’AAGCCGAAGGTGCCGATGGTCAT 3’), PCR conditions consisted in 5’ at 94°C, 35 cycles of 45” at 94°C, 30” at 52°C and 1’15” at 72°C, and a final extension of 10’ at 72°C. For the second pair, IntrFut1 (5’ ACTGGATGTCGGAGGAGTA 3') and Fut1STOP (5' CTATCCCAGAAATCTCCAGGGC 3'), the conditions were: 3’ at 98°C, 40 cycles of 30” at 98°C, 20” at 52°C and 40” at 72°C, and a final extension of 5’ at 72°C. For *Fut2* the PCR consisted in 3’ at 98°C, followed by 40 cycles of 30” at 98°C, 20” at 58°C and 1’ at 72°C and a final extension of 5’ at 72°C with primers rFut2.3 (5’ATGAGCACCGCCCAGGTCCCCTTC 3’) and rFut2.2inv (5’ TCAGTGCTTGAGCAATGGGGACAG 3’). PCR conditions for amplification of *Sec1* were the same as for *Fut2*, except the annealing temperature (56°C); primers used were Sec1.3 (5' ATGAGATTCGCCCCTGACTATGTCC 3') and Sec1.2inv (5' CTAGAGGCCACTCCACAAGGC 3'). PCR products were sequenced on an automatic sequencer ABI PRISM 310 Genetic Analyzer (PE Applied Biosystems) using the amplification primers. Sequences were submitted to GenBank (see S1 Table for accession numbers).

Sequences were aligned using BioEdit software version 7.0.9 [60]. Since these genes are highly divergent in the transmembrane domain and in the stem region, only their catalytic domain was considered and tested for gene conversion by using GARD, as implemented in the Datamonkey Web Server [61,62]. All the analyses were performed considering three distinct groups (all mammals, all mammals except leporids and leporids only) and a general reversible process model (REV) with beta-gamma variation and two rate classes. GARD infers the number of recombination breakpoints (b) by starting with b = 0 and increasing b in increments of one until the corrected Akaike Information Criteria (AICc) score does not improve further [63]. For each dataset, neighbor-joining (NJ) trees were constructed for each segment identified by GARD. Trees were estimated with software MEGA version 5.1 [64] under the Tamura 3-parameter model with the following parameters: 10,000 replicates, bootstrap method, gamma distributed rates and partial deletion.

### Statistical analyses

Analysis of the frequencies of *Fut2* promoter variant alleles between the survivors and dead animals in the Chèvreloup population was performed by a likelihood ratio Chi-square test. Comparisons of RNA levels between the *Sec1* and/or *Fut1* and *Fut2* genes in the duodenum of rabbits was performed using a Mann-Whitney test. The two-tailed Fisher’s exact test was used to compare groups of rabbits with *Sec1* mRNA expression within/above or below the *Fut1* and *Fut2* range of expression (high or low Sec1 mRNA expressors, respectively) to groups above or below median values of virus binding to duodenum extracts (high or low virus binders, respectively). Mean values of positive cells obtained by flow cytometry from independent experiments were compared by a Student’s t-test.

## Supporting Information

S1 Fig Alignment of the catalytic domain of α1,2fucosyltransferases for 23 species illustrating the gene conversion events. For each mammalian species the 3 enzymes are shown. Dark grey: positions that differ in more than one gene relatively to *Xenopus tropicalis*. Light grey: positions that are identical in more than one gene between the positions identified by dark grey. Black: premature stop codons. Dots mean identity with the *X*. *tropicalis* sequence.? indicate positions that were ambiguous upon sequencing. The alignment comprises amino acid positions 254 to 354 of *Homo sapiens* FUT1. Full names of each species and accession numbers of sequences are given in Supplementary Table 1.
(TIF)

S2 Fig Neighbour-joining (NJ) trees of the α1,2fucosyltransferase genes *Fut1*, *Fut2* and *Sec1*. NJ tree of the entire catalytic domain including all mammals except leporids (A); NJ trees of segment 1 defined by GARD (nucleotides 235–609 of *Homo sapiens FUT1*) (B) and segment 2 (nucleotides 610–1095 of *Homo sapiens FUT1*) (C). NJ trees of the entire catalytic domain of leporids and of segments 1 and 2 defined by GARD, respectively (nucleotides 235–735 and 736–1119 of *Oryctolagus cuniculus Fut1*) (D; E; F).
(DOC)

S1 Table List of sequences retrieved from GenBank and Ensembl or obtained from this study. (DOC)
