# Supplementary figures and images for: Neofunctionalization of the Sec1 α1,2fucosyltransferase Paralogue in Leporids Contributes to Glycan Polymorphism and Resistance to Rabbit Hemorrhagic Disease Virus
Source: PLoS Pathog. 2015 Apr 15;11(4):e1004759. doi: 10.1371/journal.ppat.1004759 (PMC4398370; doi:10.1371/journal.ppat.1004759)

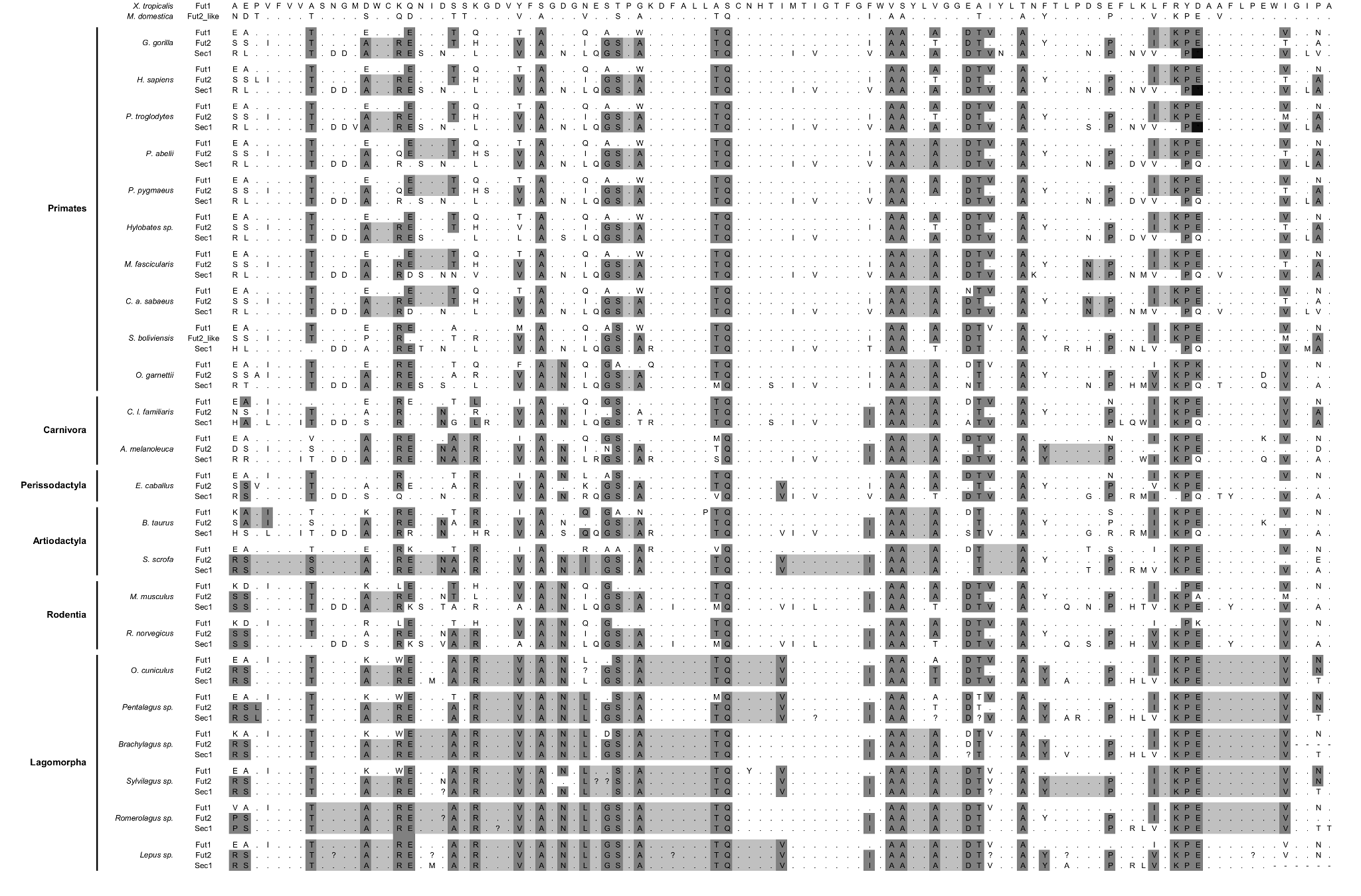

Supplement: S1 Fig — For each mammalian species the 3 enzymes are shown. Dark grey: positions that differ in more than one gene relatively to Xenopus tropicalis. Light grey: positions that are identical in more than one gene between the positions identified by dark grey. Black: premature stop codons. Dots mean identity with the X. tropicalis sequence.? indicate positions that were ambiguous upon sequencing. The alignment comprises amino acid positions 254 to 354 of Homo sapiens FUT1. Full names of each species and accession numbers of sequences are given in Supplementary Table 1. (TIF) [file ppat.1004759.s001.tif]
