# Supplementary material for: Neofunctionalization of the Sec1 α1,2fucosyltransferase Paralogue in Leporids Contributes to Glycan Polymorphism and Resistance to Rabbit Hemorrhagic Disease Virus
Source: PLoS Pathog. 2015 Apr 15;11(4):e1004759. doi: 10.1371/journal.ppat.1004759 (PMC4398370; doi:10.1371/journal.ppat.1004759)
Supplement: S2 Fig — NJ tree of the entire catalytic domain including all mammals except leporids (A); NJ trees of segment 1 defined by GARD (nucleotides 235–609 of Homo sapiens FUT1) (B) and segment 2 (nucleotides 610–1095 of Homo sapiens FUT1) (C). NJ trees of the entire catalytic domain of leporids and of segments 1 and 2 defined by GARD, respectively (nucleotides 235–735 and 736–1119 of Oryctolagus cuniculus Fut1) (D; E; F). (DOC) [file ppat.1004759.s002.doc]

**
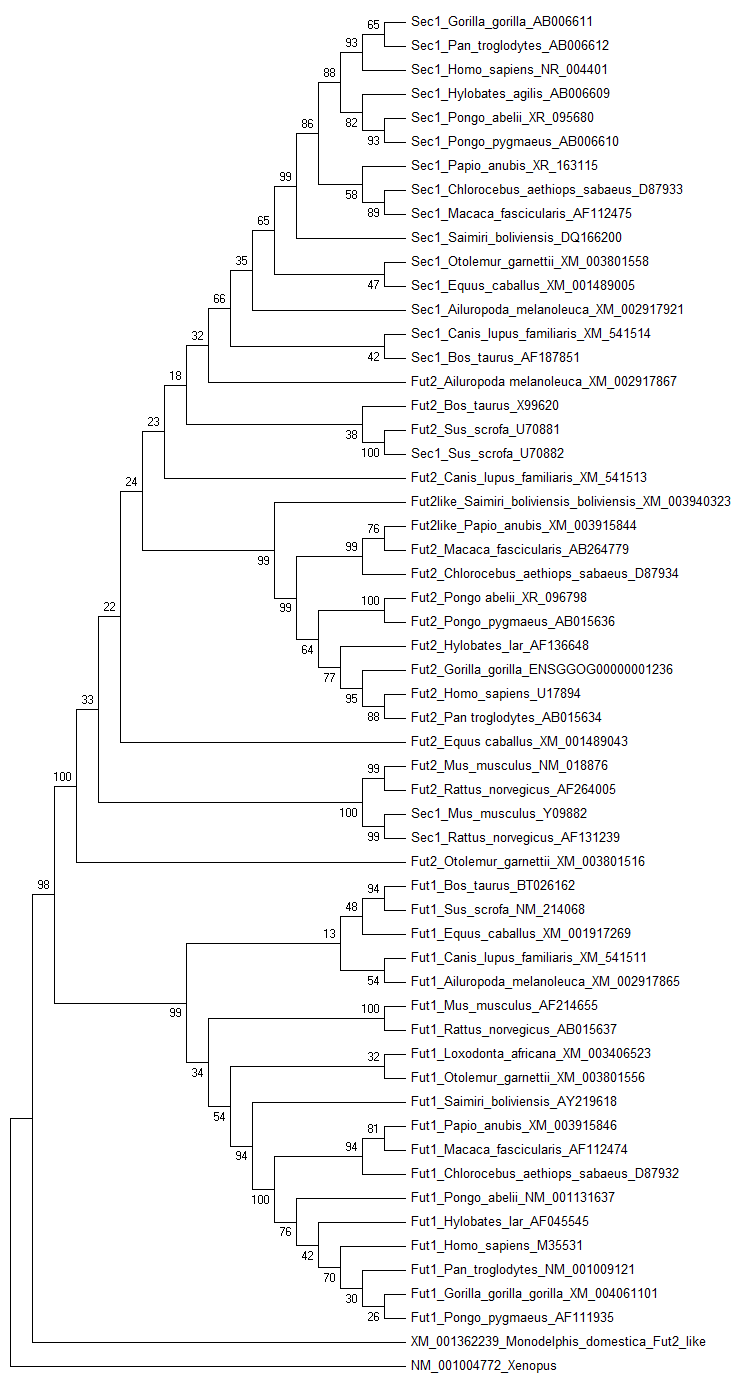
**

**A**

Sec1

Fut2

Fut1

**
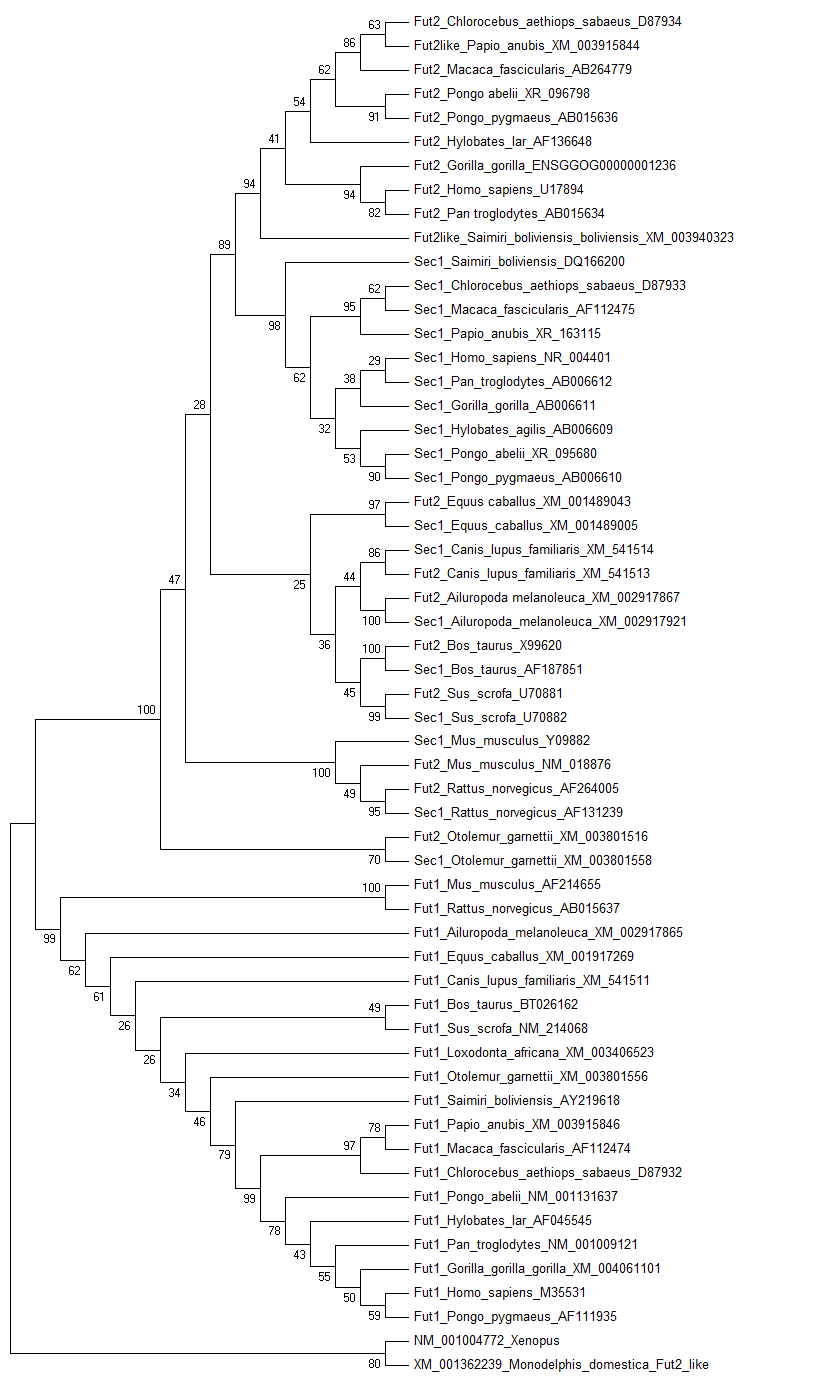
**

Fut1

Fut2

**B**

Sec1

Fut2+Sec1

**
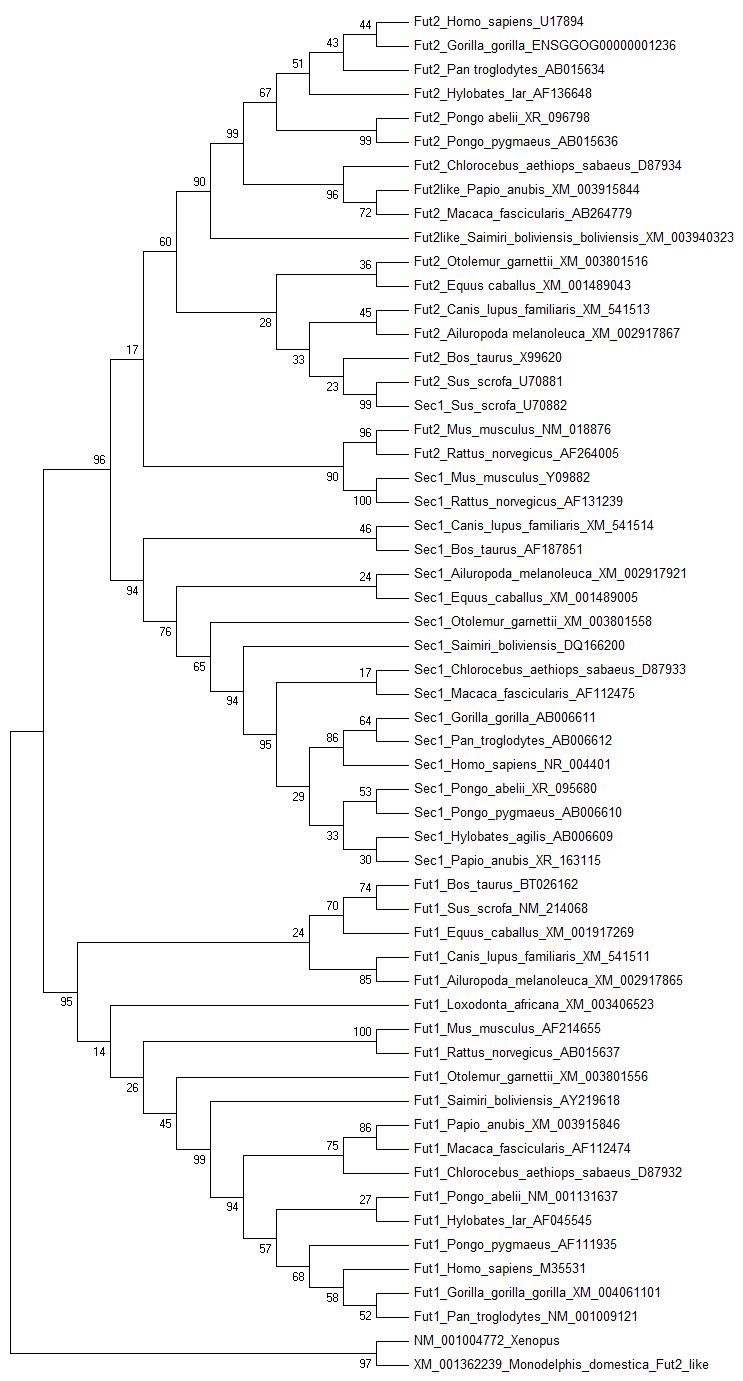
**

**C**

Fut2

Fut2+Sec1

Sec1

Fut1

**
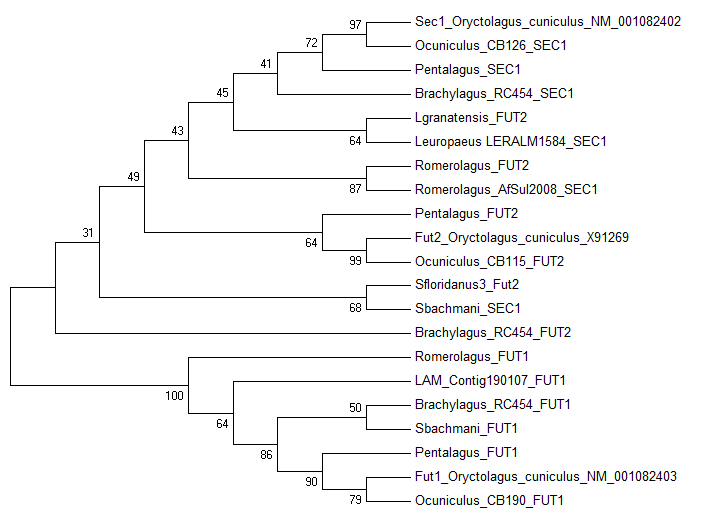
**

**D**

Sec1

Fut2+Sec1

Fut2

Fut1

**
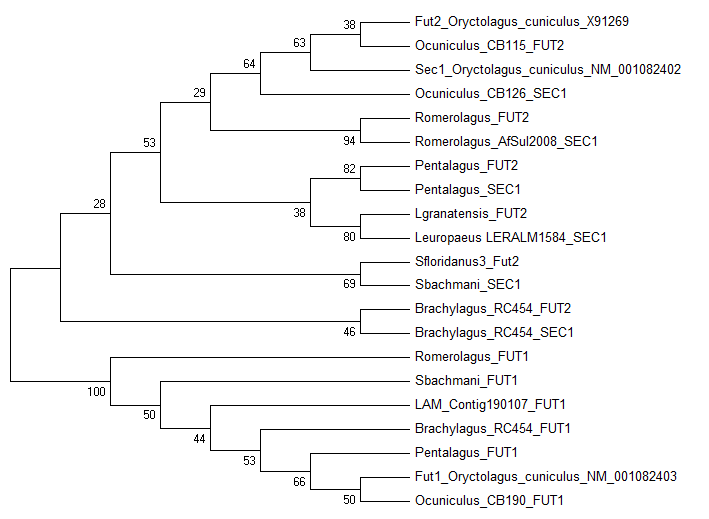
**

Fut1

**E**

Fut2+Sec1

**
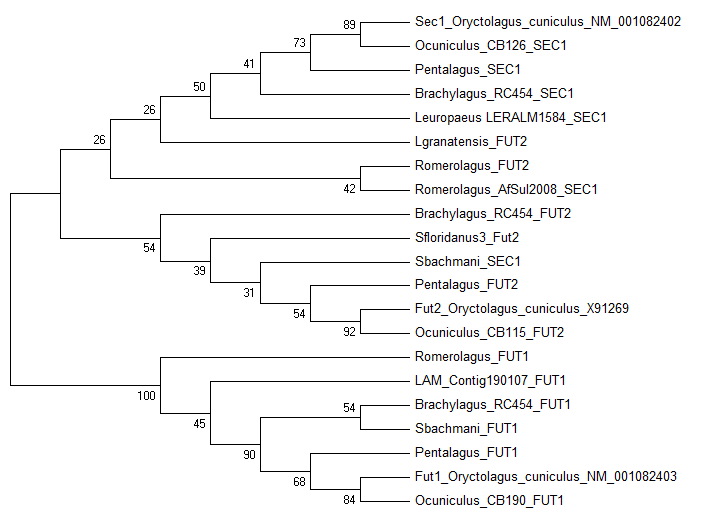
**

**F**

Sec1

Fut2+Sec1

Fut2

Fut1
