# Supplementary material for: Neofunctionalization of the Sec1 α1,2fucosyltransferase Paralogue in Leporids Contributes to Glycan Polymorphism and Resistance to Rabbit Hemorrhagic Disease Virus
Source: PLoS Pathog. 2015 Apr 15;11(4):e1004759. doi: 10.1371/journal.ppat.1004759 (PMC4398370; doi:10.1371/journal.ppat.1004759)
Supplement: S1 Table — (DOC) [file ppat.1004759.s003.doc]

**Table S1.** List of sequences used in the study and retrieved from GenBank and Ensembl.

| **Species name** | **Gene** | **GenBank accession number/Ensembl** |
| --- | --- | --- |
| *Homo sapiens* | *Fut1* | M35531 |
|  | *Fut2* | U17894 |
|  | *Sec1* | NR_004401 |
| *Pongo abelii* | *Fut1* | Scaffold [19:50290148-50295321:-1](http://www.ensembl.org/Pongo_abelii/Location/View?r=19:50290148-50295321:-1;g=ENSPPYG00000010213) |
|  | *Fut2* | XR_096798 |
|  | *Sec1* | XR_095680 |
| *Gorilla gorilla* | *Fut1* | XM_004061101 |
|  | *Fut2* | AF080606 |
|  | *Sec1* | AB006611 |
| *Hylobates lar* | *Fut1* | AF045545 |
|  | *Fut2* | AF136648 |
| *Hylobates agilis* | *Sec1* | AB006609 |
| *Chlorocebus aethiops sabaeus* | *Fut1* | D87932 |
|  | *Fut2* | D87934 |
|  | *Sec1* | D87933 |
| *Pongo pygmaeus* | *Fut1* | AF111935 |
|  | *Fut2* | AB015636 |
|  | *Sec1* | AB006610 |
| *Otolemur garnettii* | *Fut1* | XM_003801556 |
|  | *Fut2* | XM_003801516 |
|  | *Sec1* | XM_003801558 |
| *Pan troglodytes* | *Fut1* | NM_001009121 |
|  | *Fut2* | AB015634 |
|  | *Sec1* | AB006612 |
| *Saimiri boliviensis* | *Fut1* | AY219618 |
|  | *Fut2*like | XM_003940323 |
|  | *Sec1* | DQ166200 |
| *Papio anubis* | *Fut1* | XM_003915846 |
|  | *Fut2* | XM_003915844 |
|  | *Sec1* | XR_163115 |
| *Macaca fascicularis* | *Fut1* | AF112474 |
|  | *Fut2* | AB264779 |
|  | *Sec1* | AF112475 |
| *Canis lupus familiaris* | *Fut1* | XM_541511 |
|  | *Fut2* | XM_541513 |
|  | *Sec1* | XM_541514 |
| *Ailuropoda melanoleuca* | *Fut1* | XM_002917865 |
|  | *Fut2* | XM_002917867 |
|  | *Sec1* | XM_002917921 |
| *Equus caballus* | *Fut1* | XM_001917269 |
|  | *Fut2* | XM_001489043 |
|  | *Sec1* | XM_001489005 |
| *Bos taurus* | *Fut1* | BT026162 |
|  | *Fut2* | X99620 |
|  | *Sec1* | AF187851 |
| *Sus scrofa* | *Fut1* | NM_214068 |
|  | *Fut2* | U70881 |
|  | *Sec1* | U70882 |
| *Mus musculus* | *Fut1* | AF214655 |
|  | *Fut2* | NM_018876 |
|  | *Sec1* | Y09882 |
| *Rattus norvegicus* | *Fut1* | AB015637 |
|  | *Fut2* | AF264005 |
|  | *Sec1* | AF131239 |
| *Oryctolagus cuniculus* | *Fut1* | X80226 |
|  | *Fut2* | X91269 |
|  | *Sec1* | X80225 |
| *Pentalagus sp.* | *Fut1* | KP331800 |
|  | *Fut2* | KP331790 |
|  | *Sec1* | KP331795 |
| *Brachylagus sp.* | *Fut1* | KP331801 |
|  | *Fut2* | KP331791 |
|  | *Sec1* | KP331796 |
| *Sylvilagus sp.* | *Fut1* | KP331802 |
|  | *Fut2* | KP331792 |
|  | *Sec1* | KP331797 |
| *Romerolagus sp.* | *Fut1* | KP331803 |
|  | *Fut2* | KP331793 |
|  | *Sec1* | KP331798 |
| *Lepus sp.* | *Fut1* | KP331804 |
|  | *Fut2* | KP331794 |
|  | *Sec1* | KP331799 |
| *Loxodonta africana* | *Fut1* | XM_003406523 |
| *Monodelphis domestica* | *Fut2*like | XM_001362239 |
| *Xenopus tropicalis* | *Fut1* | NM_001004772 |
